# Randomized trial of acupoints herbal patching in Sanfu Days for asthma in clinical remission stage

**DOI:** 10.1186/s40169-016-0084-7

**Published:** 2016-02-04

**Authors:** Libing Zhu, Wei Zhang, Vivian Wong, Ziea Eric, Lixing Lao, Kwaiching Lo, Waichung Chan, To Yau, Lei Li

**Affiliations:** School of Chinese Medicine, The University of Hong Kong, 10 Sassoon Road, Pokful, Hong Kong, China; Chinese Medicine Department, Hospital Authority, Hong Kong, China; Center of Reproductive Medicine and Constitution of Traditional Chinese Medicine, Beijing University of Chinese Medicine, Beijing, China

**Keywords:** Asthma, Randomized trial, Acupoints herbal patching, Sanfu days

## Abstract

**Background:**

Although China has a long history of using acupoints herbal patching (acupoints herbal patching means applying herbal patch on special acupoints to stimulate skin to form blisters, hyperemia, and even suppuration) in Sanfu Days (Sanfu Days are supposed to be the three hottest days in a year which is calculated by the ancient calendar) for the treatment of asthma, there is insufficient evidence to support its effectiveness and safety issues. This study investigated the efficacy and safety of acupoints herbal patching compared with placebo in participants with asthma in clinical remission stage.

**Methods:**

We enrolled participants with asthma in clinical remission stage, above 13 years old and both genders in a randomized, double-blind and placebo-control trial at clinical center, School of Chinese Medicine, The University of Hong Kong to assess the effectiveness and safety of acupoints herbal patching, as compared with placebo, when added to guidelines-based therapy. The trial was conducted for three times (these three times were 19 July, 29 July and 8 August 2010), and the primary outcome was pulmonary function test. Secondary outcome was self-made questionnaire which were designed based on Traditional Chinese Medicine theory and clinical experience summary.

**Results:**

Three hundred and twenty three eligible participants were enrolled, they were randomly assigned to acupoints herbal patching group (n = 165), placebo control group (n = 158). There was no significant difference in primary and secondary outcome as compared with placebo group at the end of 3rd treatment and four times follow ups. But sub-analysis of secondary outcome in four times follow ups showed that acupoints herbal patching significantly reduced the proportion of participants who didn’t need medical treatment when they had a small rise in asthma-related symptoms increased from 6–15 % at 1st follow up and 0–7 % at 3rd follow up (P < 0.05). It indicated that the proportion of participants who can spontaneous resolution of an asthma attack increased through acupoints herbal patching. In addition, acupoints herbal patching was significantly superior to placebo in reducing the percentage of participants who were susceptibly waken up by asthma symptoms from 27–14 %, and the percentage of participants who had the symptom of running nose and sneezing before onset from 18–8 % at 2nd follow up (P < 0.05). Improvements also occurred with treatment group, it reduced the proportion of participants who were spontaneous sweating at 3rd follow up (P < 0.05).

**Conclusions:**

There was no significant difference between acupoints herbal patching and placebo in pulmonary function test in this study. Self-made questionnaire showed that the lasting effect of acupoints herbal patching was significantly better than placebo in reducing the need for medications to control asthma and the proportion of susceptible symptoms in participants with asthma in clinical remission stage. It showed that the low quality of life caused by asthma-related symptoms was significantly improved through acupoints herbal patching in Sanfu Days. Besides, acupoints herbal patching was as safe as placebo for chronic stable asthma.

*Trial registration number*: HKUCTR-1128, *Registration date* 22 Jul 2010

## Background

Asthma is a clinical syndrome of unknown etiology that diagnosed on the basis of symptoms including wheeze, dyspnoea, and cough, etc, accompany with the objective evidence of airflow obstruction [1]. Currently, β-agonists still as the first-line drug in treating acute asthma attack and glucocorticoids used to treat chronic asthma while are associated with various adverse effects [2–10]. In china, there was a long history of using acupoints herbal patching in Sanfu Days for preventing and treating asthma. Sanfu Days are calculated by Heavenly Stems and Earthy Branches which are supposed to be the three hottest days in a year. According to the provision, the third and fourth Gengri after the summer solstice is recorded as the first and second hottest days, and the first Gengri after the beginning of autumn is recorded as the third hottest day. In these days, body’s Yang-qi (positive energy) has an exuberant trend with the aid of Yang-qi developed by nature, and the Cold-qi in the body also in the condition of easily dissolve. Acupoints herbal patching means applying medicinal cakes which are made of Chinese herbs on special acupoints. These herbs are pungent in flavor, warm in property, and have the function of dredging the channel, which can stimulate skin to form blisters, hyperemia and even suppuration. As a result, acupoints herbal patching combined with Sanfu Days get a purpose of adjusting Yin and Yang, preventing and curing diseases, and enhancing body resistance through acupoints stimulation and drug infiltration absorption. In modern terms, acupoints herbal patching is similarly to transdermal drug delivery. Many Chinese scientists have already conducted some animal and clinical studies on the efficacy of acupoints herbal patching for the treatment of asthma. So far, they found that acupoints herbal patching was effective in improving lung function and syndromes in participants with asthma not only from animal studies but also clinical studies [11–19]. But the weakness of previous clinical studies, such as poor methodologically, small sample size, short follow-up, didn’t mention whether get Institutional Review Board (IRB) approval before study, also not mentioned drop-out rate and safety issue, all these make people unconvinced of the previous findings [20–22]. Hence, this study tried to design a more rigorous, bigger sample size and longer follow-up clinical randomized control trial (RCT) aim to investigate the efficacy and safety of acupoints herbal patching compared with placebo. The hypothesis of this study was that there was no significant difference between acupoints herbal patching group and placebo group in preventing and reducing the frequency and severity of asthmatic attacks.

## Methods

### Participants

This study was a randomized, double-blind, placebo-controlled trial in 323 asthma participants in clinical remission stage (above 13 years old and in both genders). The diagnose standard of asthma in clinical remission stage means participants had a history of asthma attack in the past 12 months, but asthma-related signs and symptoms disappeared, pulmonary function recover to the level before acute asthma attack and maintain more than 3 months when they entered this study. Documentation of symptoms of asthma or a physician’s diagnosis of asthma for more than 1 year preceding the study visit was required. Participants need to meet the inclusion criteria listed as follow: symptoms occurring or worsening at night, episodic symptoms of airflow obstruction; difficulty breathing, chest tightness, cough (worse at night); symptoms awakening the patient, occurring or worsening with exercise; viral infections, and changes in weather.

Participants would be ineligibility if they meet one or more following exclusion criteria: acute asthma attack, fever and pharyngitis, pregnancy, tuberculosis, severe cardiac and pulmonary diseases. Participants were also excluded if they had diabetes mellitus, allergy to topical medication and hypersensitive skin condition. Additional exclusion criteria were bleeding disorders, severe heart diseases or with pacemaker and keloid.

The study protocol (21006030HA) was approved by the Institutional Review Board of the University of Hong Kong/Hospital Authority Hong Kong West Cluster (HKU/HA HKWC IRB) with coding UW 10-274. This clinical trial was authorized by Clinical Trials Centre of The University of Hong Kong (HKUCTR-1128). URL of register can be found in http://www.hkuctr.com/Study/Show/f5d04b243cb14c25b9033ec55e96f6f5. Grants from Hospital Authority (No.200006030). All study participants need to provide written informed consent, those younger than 18 years of ages were also required to provide assent and then signature by their parent or legal guardian.

### Procedures

After screening visit, enrolled participants were randomly assigned to an acupoints herbal patching group and a placebo control group by a third person (Wang ZH) and she did not practice throughout the study. Random numbers were distributed by the formula of [= rand ()] in Microsoft Office Excel 2007 (fraction between 0 and 1), and if the produced random number ≥0.5, it was assigned to acupoints herbal patching group, and otherwise, it was belonged to placebo group. The principal investigator of this study—Dr. Li L, who obtained Ph.D. degree of acupuncture and moxibustion and have more than 30 years acupuncture clinical experience. Besides he is not only the registered Chinese Medicine practitioner in Mainland China, but also Hong Kong registered Chinese Medicine practitioner. The rest practitioners who participated in this study all have bachelor or bachelor above degree of Traditional Chinese Medicine (TCM), and all of them are registered Chinese Medicine practitioners.

A study coordinator (Choi PY) allocated the random numbers to participants. Regarding as the allocation results, they were sealed into opaque envelopes and allocation concealment must be warranted. Totally twelve medical beds were provided during these three treatment days and treatment hours were from 9 a.m. to 7 p.m. During the treatment days, one usher (totally ten ushers who were Li XZ, Ching WT, Yau T, Zhang WD, Tse WM, Wu TT, Kan WY, Lo MF, Shi HF and Irene) guided one patient to the reception room, and then the receptionist (totally two receptionist: Leung LF and Lam CK) checked the random numbers list to decide which kind patch was given for this participant and told the result to taking patch person (totally two taking patch person: Lam WC and Chia KL). Then taking patch person took a sealed box with eleven patches inside to plaster (totally 10 plasters: Li L, Chan WC, Lo KC, Zhang W, Eric Z, Wong V, Tin PY, Chan HN, Cheung SK and Choi WF). Plaster spent nearly 5 min to paste patches in acupoints (totally 11 acupoints, and one patch for one acupoints), and then plaster used approximately 4 cm × 4 cm hypoallergenic tape (3 M™ Micropore™ Tape 1535-3) to stick patches in skin. Then the usher took the participant to waiting room, research assistants (Wu FM and Cheung CY) in waiting room removed the patches after 2 h. Last, participants can leave treatment place if they didn’t have any uncomfortable. During the whole process, nobody has idea of which treatment they have been received or given except Wang ZH. Until final statistic finished, Wang ZH managed unblinding and the random code was broken.

**Fig. 1:**
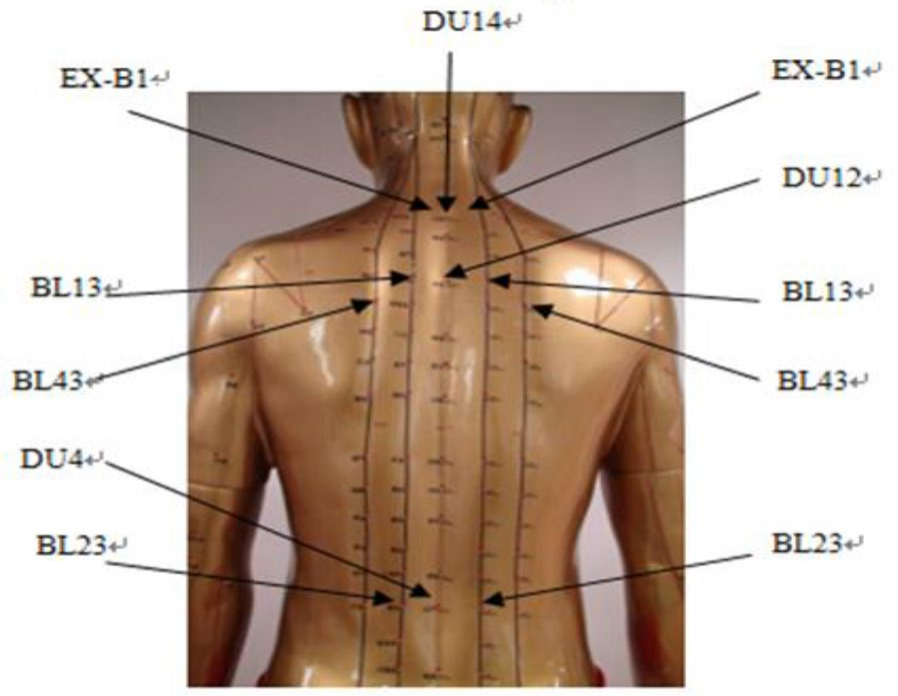
Acupoints

**Fig. 2:**
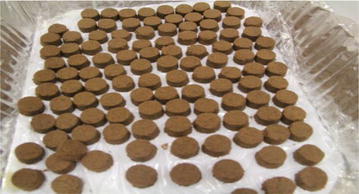
Herbal patch

**Fig. 3:**
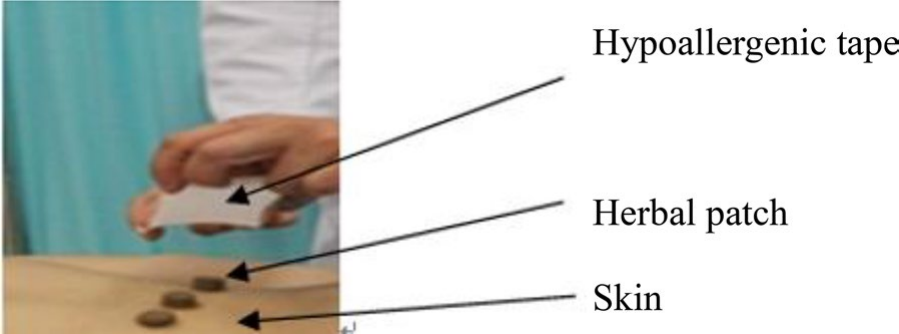
Acupoints herbal patching schematic diagram

Both the acupoints herbal patching and placebo treatment was consisted of three sessions with 2 h duration (these three sessions were 19 July, 29 July and 8 August 2010). For all participants, 11 acupoints were applied by patches: Du14, Du12, Du4, BL13 (both side), BL23 (both side), BL43 (both side) and EX-B1 (both side) (Fig. 1). In both groups, every selected acupoints was applied one patch and every patch was about 2 g (Fig. 2). The final formula used in treatment group was a combination of the record in the “*Zhang’ Medicine*” and the formula frequency used in clinical trials. In the acupoints herbal patching group, the selected herbals were Sinapi Alba, Radix Corydalis Yanhusuo, Processed Euphorbia kansui, Asari Herba cum Radice, Ephedrae Herba, Processed Radix Aconiti Praeparata, Cinnamomum cassia and Eugenia caryophyllata, with a proportion in 2:1:1:1:1:1:1:1. At last, these herbals were mixed together with ginger juice. In placebo group, the selected materials with a proportion of 3:3:1 including pure starch, red rice and black glutinous rice, which finally were mixed together by petroleum jelly (Fig. 3).

**Fig. 4:**
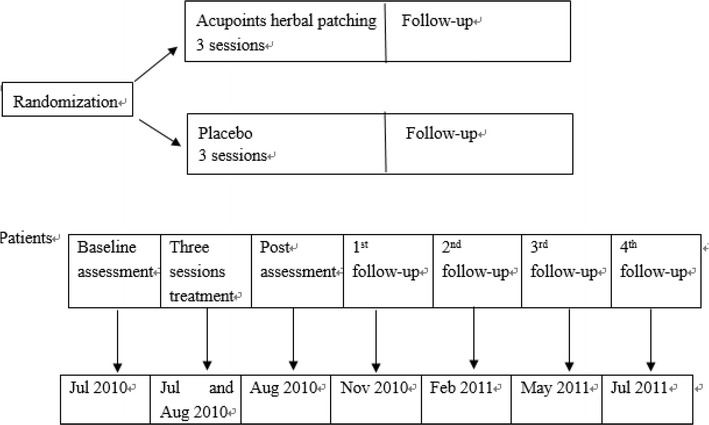
Study design

All participants completed the self-made questionnaires and pulmonary function test at baseline, the end of 3rd treatment, and four times follow ups (Fig. 4). Questionnaires were assessed by physicians (Li L, Chan WC, Lo KC, Zhang W, Eric Z, Wong V, Tin PY, Chan HN, Cheung SK and Choi WF) through face to face. The primary outcome was pulmonary function test: forced expiratory volume in one second (FEV1) and forced expiratory volume in one second/forced vital capacity [FEV1/FVC (%)], the pulmonary function test machines were provided by the lab in School of Chinese Medicine, The University of Hong Kong and all operators didn’t know they do lung function test for which group participants. Secondary outcome was self-made questionnaire which included the number of days with asthma-related symptoms, asthma-related health care use, the frequency of bronchodilator used during asthma attack, the number of asthma related symptoms which were associated with Chinese Medicine and the percentage of participants with twenty-three specific such symptoms. These twenty-three symptoms were shown in Table 2.

### Statistical analysis

The participants who received at least baseline assessment constituted the intention-to-treat (ITT) analysis, with a two-sided alpha level of 0.05. For all primary and secondary outcome measures, sensitivity analyses were performed by replacing missing data with the last value. An additional per-protocol analysis (PP) was carried out for participants without major protocol violation who could be assessed at the end of 3rd treatment. Statistical analyses were performed with the Statistical Package for the Social Sciences (SPSS) software program (version 19) for Windows XP. The analysis included data from week 0 (baseline) through week 8 (at the end of 3rd treatment) and was performed with the use of an analysis of covariance (ANCOVA), group (acupoints herbal patching group and placebo group) as a fixed factor and time as covariance (baseline as the 1st time, post-treatment as the 2nd time). ANCOVA was conducted for continuous variables such as the number of asthma related symptoms which were associated with Chinese Medicine, the number of days with asthma-related symptoms and pulmonary function test: FEV and FEV1/FVC (%) [23]. Binary logistic regression was conducted for the categorical variable such as asthma-related health care and the percentage of participants with twenty-three specific such symptoms [24]. Non-parametric test was conducted for a ranked variable such as the frequency of bronchodilator used during asthma attack. For the 1st, 2nd 3rd and 4th follow up measurements, two-sided t tests and Chi-square tests were done for groups’ pair wise comparisons [23]. Sample size was calculated according to the effective rate that conducted by previous clinical trials of acupoints herbal patch for asthma. The no. of target subjects to be recruited at local site was 380 (190 per treatment group × 2 groups) through statistic.

## Results

### Enrollment

**Fig. 5:**
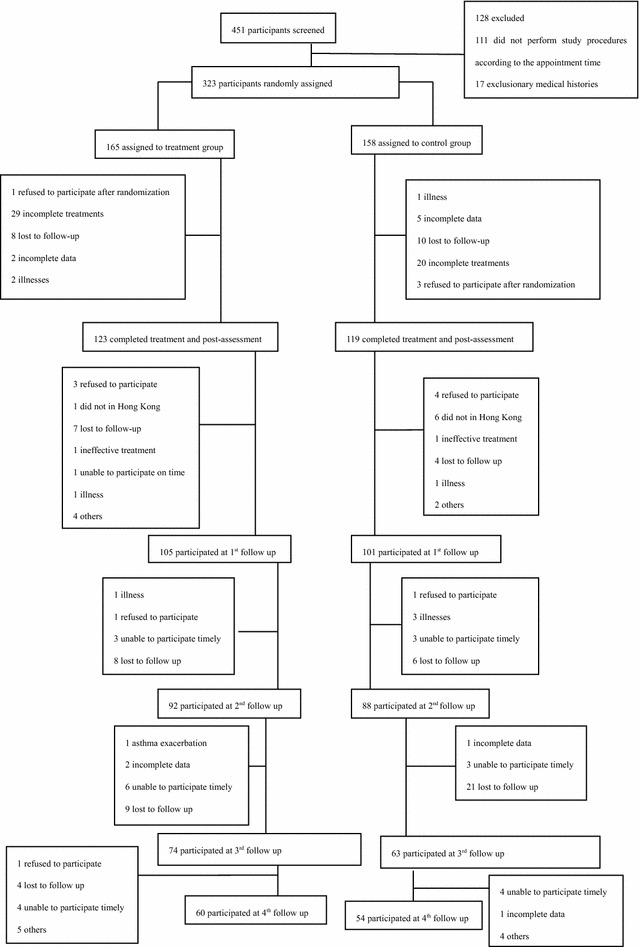
Trial flow

Initially, 451 participants with asthma in clinical remission stage applied to participate in this project. Figure 5 shows the trial flow. After screened, 323 participants underwent randomization: 165 to acupoints herbal patching group and 158 to placebo group. All subjects (323) were included in the ITT population. Forty-two participants in acupoints herbal patching group and thirty-nine participants in placebo group stopped the treatment prematurely. A PP analysis was conducted for the 242 subjects who could be assessed at the end of the 3rd treatment.

Tables 1, 2 showed the baseline characteristics of participants in the two study groups and their characteristics were basically similar: for both groups, the average age was 44 years old, 44 % were male, and the average disease course of asthma were 22.74 ± 22.68 year in placebo group and 21.13 ± 21.08 year in acupoints herbal patching group. At enrollment, regarding the number of days on which subjects had asthma-related symptoms in acupoints herbal patching group was 10.25 ± 12.26 and 7.97 ± 10.94 in the placebo group. The mean (±SD) FEV_1_ was 2.13 ± 1.08 in acupoints herbal patching group and 2.07 ± 1.00 in placebo group, and the mean ratio of FEV_1_ to FVC was 85.97 ± 13.85 in acupoints herbal patching group and 86.25 ± 13.93 in placebo group. Regarding the health care used when they had a small rise in asthma-related symptoms, no difference occurred in treatment group and control group. Besides, there was nothing different between acupoints herbal patching group and placebo group in the distribution of the frequency of bronchodilator used during asthma attack in previous 1 year. Additionally, the number of symptoms which were associated with Chinese Medicine was also nearly same (9.12 ± 4.28 in TG, 9.22 ± 4.18 in CG). There was also nothing different in percentage of participants with twenty-three specific such symptoms between acupoints herbal patching group and placebo group.

**Table 1:** Characteristics of study participants before and after treatment

Variable	Before treatment			After treatment		
	Acupoints herbal patching mean (SD)	Placebo mean (SD)	P	Acupoints herbal patching mean (SD)	Placebo mean (SD)	P
Age–year	44.28 ± 17.15	44.53 ± 17.83	0.90			
Gender—no. (%)						
Male	73 (44.24)	69 (43.67)	0.92			
Female	92 (55.76)	89 (56.32)				
Duration of asthma—year	21.13 ± 21.08	22.74 ± 22.68	0.51			
Asthma-related symptoms—no. of days in previous 1 year^a^	10.25 ± 12.26	7.97 ± 10.94	0.08			
Asthma-related symptoms—no. of days during the treatment				2.58 ± 0.47	2.68 ± 0.48	0.88
Lung function						
FEV_1_	2.13 ± 1.08	2.07 ± 1.00	0.57	2.04 ± 0.41	2.12 ± 0.42	0.14
FEV_1_: FVC × 100	85.97 ± 13.85	86.25 ± 13.93	0.86	87.36 ± 0.66	87.20 ± 0.67	0.87
Asthma-related health care use—no. (%)						
≥1 admit to A&E^b^	48 (29)	46 (29)	1.00	4 (3)	3 (3)	0.73
≥1 hospitalization	26 (16)	29 (18)	0.54	3 (2)	2 (2)	0.68
≥1 outpatient visit	134 (81)	134 (84)	0.39	13(12)	14 (12)	0.77
≥1 prescription	139 (84)	136 (86)	0.64	65 (53)	57 (48)	0.44
≥1 by own medication	34 (21)	24 (15)	0.21	2 (2)	2 (2)	0.97
≥1 didn’t need treatment	26 (16)	17 (11)	0.19	8 (7)	6 (5)	0.63
The frequency of bronchodilator during asthma attack—no. (%)			0.21			0.72
More than twice per day	37 (22)	29 (18)		13 (11)	13 (11)	
Once to twice per day	50 (30)	62 (39)		34 (28)	33 (28)	
2–3 times per week	12 (7)	18 (11)		3 (2)	2 (2)	
Less than once per week	48 (29)	36 (23)		13 (11)	7 (6)	
Never	18 (11)	13 (8)		60 (49)	64 (54)	
Symptoms which frequently appeared in asthma participants—no. of symptoms^c^	9.12 ± 4.28	9.22 ± 4.18	0.83	3.92 ± 0.34	4.01 ± 0.37	0.85

Plus-minus value are mean ± SD. P values for the comparison of means and percentages were calculated with the use of Chi-square test for categorical variables and the independent-samples test for continuous variables. FEV_1_, FVC

^a^The number of days with symptoms was calculated as the largest of the following variables during the previous 1 month: the number of days with wheezing, cough, chest tightness, or nighttime sleep disruption. This symptom scale ranges from 0–30 days

^b^Accident and emergency departments (A and E)

^c^The total number of symptoms which frequently appeared in asthma participants are 23

**Table 2:** Characteristics of study participants’ symptoms scale before and after treatment

Symptoms scale	Before treatment			After treatment		
	Acupoints herbal patching	Placebo	P	Acupoints herbal patching	Placebo	P
Detail symptoms—no. (%)						
1. Wind intolerance	61 (37)	67 (42)	0.32	13 (11)	13 (11)	0.93
2. Susceptible cold	88 (53)	96 (61)	0.09	16 (13)	10 (8)	0.25
3. Sneeze, running nose before onset	78 (47)	84 (53)	0.29	11 (9)	12 (10)	0.76
4. Onset during quarter turn	122 (74)	114 (72)	0.72	6 (5)	7 (6)	0.73
5. Rapid or difficult breathing	120 (73)	116 (73)	0.89	33 (27)	39 (33)	0.31
6. Wake up by asthma symptoms	113 (68)	110 (70)	0.83	29 (24)	23 (19)	0.42
7. Bluish complexion	24 (15)	17 (11)	0.31	4 (3)	1 (1)	0.19
8. Spontaneous sweating	52 (32)	56 (35)	0.45	6 (5)	10 (8)	0.27
9. Lassitude	95 (58)	86 (54)	0.57	20 (16)	20 (17)	0.91
10. Lack of speech	45 (27)	41 (26)	0.79	9 (7)	8 (7)	0.86
11. Decline in physical strength	79 (48)	66 (42)	0.27	19 (15)	12 (10)	0.21
12. Reduction of exercise	41 (25)	33 (21)	0.40	6 (5)	5 (4)	0.80
13. Lack of strength	91 (55)	90 (57)	0.74	23 (19)	17 (14)	0.36
14. Lack of energy after asthma attack	50 (30)	42 (27)	0.46	6 (5)	5 (4)	0.80
15. Poor appetite	31 (19)	25 (16)	0.48	4 (3)	7 (6)	0.33
16. Abdomen fullness	44 (27)	37 (23)	0.50	5 (4)	6 (5)	0.72
17. Sloopy stool	22 (13)	32 (20)	0.10	5 (4)	6 (5)	0.72
18. Diarrhea after intake of oil food	29 (18)	31 (20)	0.64	3 (2)	3 (3)	0.97
19. Fear of cold	71 (43)	72 (46)	0.65	15 (12)	12 (10)	0.60
20. Soreness and weakness of waist and knees	79 (48)	85 (54)	0.29	9 (7)	15 (13)	0.17
21. Tinnitus	60 (36)	54 (34)	0.68	10 (8)	11 (9)	0.76
22. Frequent urination/night urination	52 (32)	50 (32)	0.98	7 (6)	11 (9)	0.29
23. Redness, hotness or anxiety	58 (35)	53 (34)	0.76	9 (7)	7 (6)	0.65

### Response to intervention

**Fig. 6:**
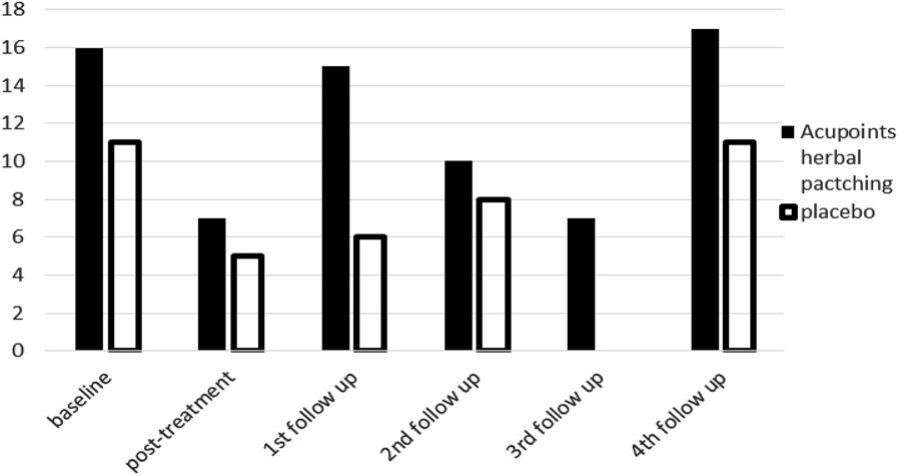
The percentage of participants who didn’t need medical treatment when they have a small rise in asthma-related symptoms. Acupoints herbal patching was better than placebo in 1st and 3rd follow up, P < 0.05

**Fig. 7:**
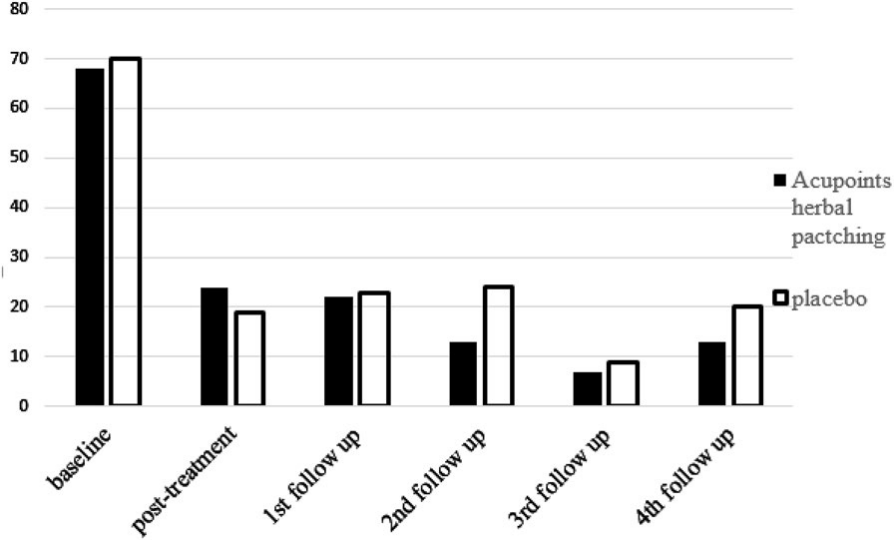
The percentage of participants suffered from waking up by asthma symptoms. Acupoints herbal patching was better than placebo in 2nd follow up, P < 0.05

**Fig. 8:**
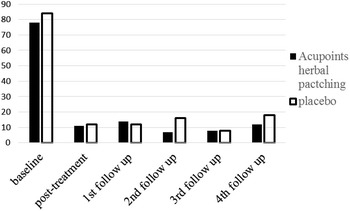
The percentage of participants suffered from sneeze, running nose before asthma onset. Acupoints herbal patching was significantly better than placebo in 2nd follow up, P < 0.05

**Fig. 9:**
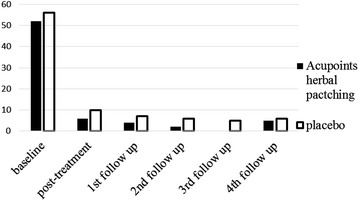
The percentage of participants suffered from spontaneous sweating. Acupoints herbal patching was better than placebo in 4th follow up, P < 0.05

**Fig. 10:**
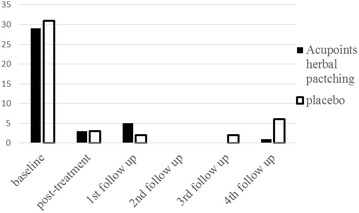
The percentage of participants susceptibly suffered from diarrhea after intake of oily food. Acupoints herbal patching was better than placebo in 4th follow up, P < 0.05

After three times acupoints herbal patching, as compared with placebo, no changes occurred in primary outcome at the end of the 3rd treatment and four times follow ups (Tables 1, 3). Also, there was no significant difference appeared in secondary outcome at the end of the 3rd treatment (Table 2). But sub-analysis of secondary outcome in the following four times follow ups was found that acupoints herbal patching was superior to placebo in improving certain symptoms (Table 4). The result of sub-analysis show that acupoints herbal patching significantly reduced the proportion of participants who didn’t need medical treatment when they had a small rise in asthma-related symptoms increased from 6–15 % compared with placebo group at the 1st follow up (Nov), and increased the proportion from 0–7 % at the 3rd follow up (May) (Fig. 6, all P < 0.05). In other words, the proportion of participants who can spontaneous resolution of an asthma attack increased through acupoints herbal patching. Additionally, acupoints herbal patching as compared with placebo group significantly reduced the percentage of participants who are susceptibly waken up by asthma symptoms from 27–14 % at 2nd follow up (Feb) (Fig. 7, P < 0.05). Similarly, acupoints herbal patching significantly reduced the proportion of participants who had the symptom of running nose and sneezing before onset from 18–8 % at 2nd follow up (Fig. 8, P < 0.05). Improvements also occurred with acupoints herbal patching group, it reduced the proportion of participants who were spontaneous sweating at 3rd follow up and diarrhea after intake of oily food at 4th follow up (Figs. 9, 10, July, all P < 0.05). All these findings showed that the low quality of life that caused by asthma-related symptoms was significantly improved through acupoints herbal patching in Sanfu Days. The top four symptoms which improved mostly in asthma participants were: susceptibly waken up by asthma symptoms and running nose, sneezing before onset which were belong to Lung-qi-deficiency; spontaneous sweating and diarrhea after intake of oily food were belong to Spleen-qi-deficiency. It showed that acupoints herbal patching has a better effect for those participants who were Lung-qi-deficiency and mainly showed as susceptibly waken up by asthma symptoms and running nose, sneezing before onset, and Spleen-qi-deficiency which mainly showed as spontaneous sweating and diarrhea after intake of oily food. Last but not least, the results of PP analysis were consistently with the ITT analysis.

**Table 3:** Primary and secondary outcomes at 1st, 2nd, 3rd, and 4th follow ups

Variable	1st follow up			2nd follow up			3rd follow up			4th follow up		
	Acupoints herbal patching mean (SD)	Placebo mean (SD)	P	Acupoints herbal patching mean (SD)	Placebo mean (SD)	P	Acupoints herbal patching mean (SD)	Placebo mean (SD)	P	Acupoints herbal patching mean (SD)	Placebo mean (SD)	P
Asthma-related symptoms—no. of days in 1 month preceding visit	4.73 ± 8.95	6.96 ± 10.91	0.11	7.71 ± 15.74	8.49 ± 10.97	0.70	6.86 ± 10.83	7.63 ± 10.06	0.67	8.55 ± 11.82	5.87 ± 9.79	0.19
Lung function												
FEV_1_	1.95 ± 0.77	1.99 ± 0.83	0.77	1.95 ± 1.02	2.03 ± 1.03	0.62	1.83 ± 0.73	1.95 ± 0.79	0.35	1.67 ± 0.65	1.89 ± 0.84	0.11
FEV_1_: FVC **×** 100	86.59 ± 13.25	87.49 ± 11.90	0.61	86.35 ± 12.90	87.10 ± 11.49	0.69	86.45 ± 12.35	88.33 ± 12.94	0.39	84.42 ± 12.97	86.56 ± 12.34	0.37
Asthma-related health care use—no. (%)												
≥1 admit to A&E	9 (9)	4 (4)	0.17	11 (12)	8 (9)	0.53	4 (5)	7 (11)	0.22	8 (13)	9 (17)	0.62
≥1 hospitalization	5 (5)	4 (4)	0.78	8 (9)	5 (7)	0.44	4 (5)	3 (5)	0.87	4 (7)	6 (11)	0.40
≥1 outpatient visit	24 (23)	29 (29)	0.34	29 (32)	30 (35)	0.71	21 (28)	21 (33)	0.53	24 (40)	27 (50)	0.28
≥1 prescription	64 (61)	68 (67)	0.34	56 (61)	64 (73)	0.09	45 (61)	44 (70)	0.27	42 (70)	42 (78)	0.35
≥1 by own medication	7 (7)	10 (10)	0.40	5 (5)	9 (10)	0.23	7 (10)	1 (2)	0.05	5 (8)	6 (11)	0.62
≥1 didn’t need treatment	16 (15)	6 (6)	0.03	9 (10)	7 (8)	0.67	5 (7)	0 (0)	0.04	10 (17)	6 (11)	0.43
The frequency of bronchodilator during asthma attack—no. (%)												
More than twice per day	22 (21)	19 (19)	0.41	26 (28)	20 (23)	0.08	13 (18)	14 (22)	0.05	14 (23)	12 (22)	0.58
Once to twice per day	32 (31)	39 (39)		21 (23)	37 (42)		20 (27)	27 (43)		30 (50)	31 (57)	
2–3 times per week	1 (1)	4 (4)		1 (1)	0 (0)		2 (3)	0 (0)		2 (3)	1 (2)	
Less than once per week	13 (12)	10 (10)		8 (9)	5 (6)		8 (11)	1 (2)		2 (3)	4 (7)	
Never	37 (35)	29 (29)		36 (39)	26 (30)		31 (42)	21 (33)		12 (20)	6 (11)	
Symptoms which frequently appeared in asthma patients—no. of symptoms	2.75 ± 2.32	2.41 ± 2.01	0.26	2.18 ± 2.10	2.18 ± 1.99	0.99	2.36 ± 2.41	2.48 ± 2.63	0.80	3.87 ± 3.36	4.94 ± 4.30	0.14

**Table 4:** Symptoms scale at 1st, 2nd, 3rd, and 4th follow ups

Symptoms scale	1st follow up			2nd follow up			3rd follow up			4th follow up		
	Acupoints herbal patching	Placebo	P	Acupoints herbal patching	Placebo	P	Acupoints herbal patching	Placebo	P	Acupoints herbal patching	placebo	P
Detail symptoms—no. (%)												
1. Wind intolerance	13 (12)	13 (13)	0.92	9 (10)	4 (5)	0.18	5 (7)	6 (10)	0.55	12 (20)	7 (13)	0.31
2. Susceptible cold	18 (17)	17 (17)	0.95	18 (20)	19 (22)	0.74	9 (12)	12 (19)	0.27	15 (25)	20 (37)	0.16
3. Sneeze, running nose before onset	14 (13)	12 (12)	0.76	7 (8)	16 (18)	0.03	8 (11)	8 (13)	0.73	12 (20)	18 (33)	0.11
4. Onset during quarter turn	19 (18)	15 (15)	0.53	11 (12)	14 (16)	0.44	7 (9)	12 (19)	0.11	19 (32)	20 (37)	0.55
5. Rapid or difficult breathing	33 (31)	22 (22)	0.12	21 (23)	19 (22)	0.84	18 (24)	17 (27)	0.72	22 (37)	27 (50)	0.15
6. Wake up by asthma symptoms	22 (21)	23 (23)	0.75	13 (14)	24 (27)	0.03	7 (9)	9 (14)	0.38	13 (22)	20 (37)	0.07
7. Bluish complexion	0 (0)	1 (1)	0.31	0 (0)	2 (2)	0.15	2 (3)	1 (2)	0.66	2 (3)	3 (6)	0.56
8. Spontaneous sweating	4 (4)	7 (7)	0.32	2 (2)	6 (7)	0.13	0 (0)	5 (8)	0.01	5 (8)	6 (11)	0.62
9. Lassitude	18 (17)	10 (10)	0.13	11 (12)	8 (9)	0.53	12 (16)	7 (11)	0.40	16 (27)	12 (22)	0.58
10. Lack of speech	5 (5)	4 (4)	0.78	6 (7)	1 (1)	0.06	2 (3)	2 (3)	0.87	6 (10)	5 (9)	0.89
11. Decline in physical strength	5 (5)	4 (4)	0.78	7 (8)	4 (5)	0.39	5 (7)	3 (5)	0.62	11 (18)	10 (19)	0.98
12. Reduction of exercise	4 (4)	0 (0)	0.05	2 (2)	0 (0)	0.16	1 (1)	2 (3)	0.47	5 (8)	3 (6)	0.56
13. Lack of strength	12 (11)	6 (6)	0.17	6 (7)	5 (6)	0.81	9 (12)	2 (3)	0.05	16 (27)	22 (41)	0.11
14. Lack of energy after asthma attack	4 (4)	4 (4)	0.96	1 (1)	2 (2)	0.53	2 (3)	2 (3)	0.87	4 (7)	6 (11)	0.40
15. Poor appetite	5 (5)	4 (5)	0.78	3 (3)	2 (2)	0.69	2 (3)	3 (5)	0.52	5 (8)	4 (7)	0.86
16.abdomen fullness	8 (8)	5 (5)	0.43	4 (4)	1 (1)	0.19	3 (4)	0 (0)	0.11	4 (7)	4 (7)	0.88
17. Sloopy stool	3 (3)	2 (2)	0.69	3 (3)	2 (2)	0.69	2 (3)	5 (8)	0.17	3 (5)	8 (15)	0.08
18. Diarrhea after intake of oil food	5 (5)	2 (2)	0.27	0 (0)	0 (0)	1.000	0 (0)	2 (3)	0.12	1 (2)	6 (11)	0.04
19. Fear of cold	10 (10)	7 (7)	0.50	5 (5)	7 (8)	0.50	10 (14)	5 (8)	0.30	8 (13)	11 (20)	0.31
20. Soreness and weakness of waist and knees	18 (17)	7 (7)	0.85	4 (4)	2 (2)	0.44	4 (5)	4 (6)	0.81	15 (25)	15 (28)	0.74
21. Tinnitus	12 (11)	8 (8)	0.40	4 (4)	7 (8)	0.31	4 (5)	1 (2)	0.24	12 (20)	12 (22)	0.77
22. Frequent urination /night urination	8 (8)	8 (8)	0.94	5 (5)	1 (1)	0.11	2 (3)	4 (6)	0.30	8 (13)	6 (11)	0.72
23. Redness, hotness or anxiety	6 (6)	6 (6)	0.95	2 (2)	4 (5)	0.38	3 (4)	2 (3)	0.80	3 (5)	6 (11)	0.23

**Table 5:** Temperature records of Sanfu Days in 2010 [22, 23]

Item time^ref^	2010				
	Maximum temperature (°C)	Temperature (°C)	Minimum temperature (°C )	Relative humidity (%)	Sun exposure (hour)
1st hottest day (July, 19) [22]	33.6	29.7	27.3	77	10.9
2nd hottest day (July, 29) [22]	31.8	28.5	24.5	83	4.6
3rd hottest day (Aug, 8) [23]	32.2	28.8	25.8	84	7.4

### Safety

Cutaneous reaction such as itching, skin warm feeling, swollen, blisters or pain were seen in almost all participants from acupoints herbal patching group. Participants in placebo group only have minor skin redness during the treatment. All these cutaneous reaction disappeared once stopped treatment. Five severe adverse events reported in treatment group: three participants appeared asthma exacerbation, one subject tended to vomited blood and automatically stopped after the 2nd acupoints herbal patching and had confirmed that has nothing to do with acupoints herbal patching, and one participants appeared the rash due to allergic to acupoints herbal patching patch. In addition, one participant has confirmed that she was pregnancy at the end of July 2010.

## Discussion

**Fig. 11:**
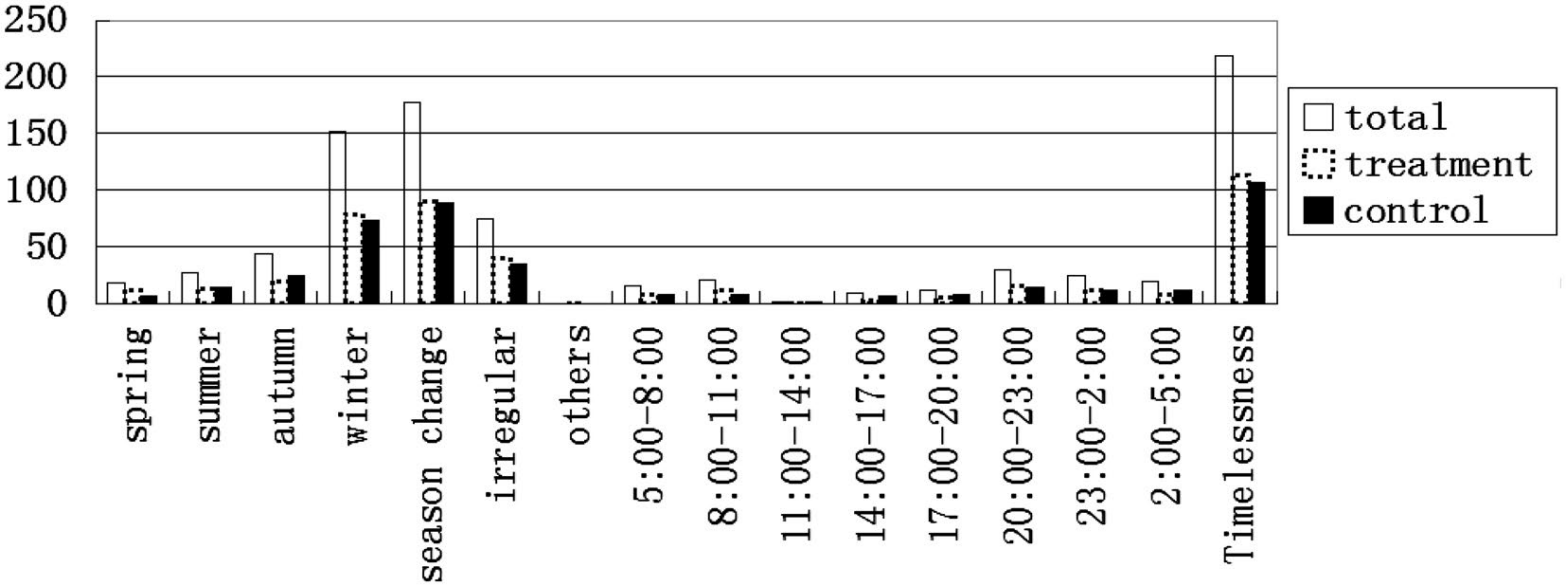
Asthma attack time distribution diagram

In this study, participants with asthma in clinical remission stage who received acupoints herbal patching had no significant difference in primary and secondary outcomes at the end of 3rd treatment comparing with placebo group. Although ANCOVA show an insignificant effect of group (P > 0.05), the effect of time was significant (P < 0.001). This indicated that both groups experienced similar improvement, including significant decrease in the number of symptoms which were associated with Chinese Medicine, the percentage of participants with twenty-three symptoms which were associated with Chinese Medicine, the number of days with asthma related symptoms, etc. Only from the result of 3rd treatment, acupoints herbal patching seemed to be equal to the function of placebo, while rethinking its concept of acupoints herbal patching in Sanfu Days which means pasting herbal patch on some special acupoints in Sanfu Days. In others words, acupoints herbal patching in Sanfu Days take effect in the treatment of asthma need three factors: the first is time (Sanfu Days), the second is meridian efficacy and the third is special herbal patch. In this study, placebo group also conducted in Sanfu Days and placebo patches were applied in same acupoints which means placebo group occupied two key factors. Hence, placebo group also can get an immediate improvement due to the psychological effect, time effect and meridian effect. So if only from the immediately result of both group at the end of 3rd treatment, there was nothing different occurred between acupoints herbal patching group and placebo group. On the whole, primary and secondary outcomes were insignificant different between treatment and control group, but the sub-analysis of secondary outcome in the 1st follow up show that the proportion of participants who did not need medical treatment when they had a small rise of asthma-related symptoms was 15 % in acupoints herbal patching group better than 6 % in placebo group (P < 0.05). Winter and seasonal change was the peak time point for asthma attack in both groups according to the asthma attacks time distribution diagram of these 323 participants (Fig. 11). The 1st follow up was conducted in November in Hong Kong and it was a time from autumn to winter when asthma was easily attacked. It indicated that treatment group participants have a stronger resistance to asthma than placebo group during season change after one course acupoints herbal patching. Besides, acupoints herbal patching was a preventive treatment for asthma.

This study is, to date, the first RCT to compare acupoints herbal patching with placebo therapy for asthma in clinical remission stage. Its strengths included big sample size, long follow-up and strict blinding. Two inevitable limitations were the self-made questionnaire and imperfect blinding. There was no doubt that this study should adopt the international accepted questionnaire for clinical trial on asthma. But this project was full of Traditional Chinese Medicine Theory characteristic so it needed a questionnaire which combined Traditional Chinese Medicine with modern medicine well. So far, there was not related questionnaire that was the reason why this study designed the first questionnaire which owned the characteristic of both Traditional Chinese Medicine and modern medicine. This questionnaire was designed based on both Chinese Medicine and Western Medicine experts’ consensus. Inevitably, this self-made questionnaire exist some shortcoming and the gap did exist between ideal and reality, such as this questionnaire only concerned about the asthma-related health care use when they had asthma-related symptoms, but didn’t provide a section to record the detailed medications participants used, and usage and dosage. Hence, future study needed to further improve the quality of this self-made questionnaire and to detect its reliability and validity. The second limitation was the imperfect blinding. Firstly, the smell of real and placebo herbal patch was different, even participants didn’t have the chance to see the patches, we cannot stop patches’ odor emitted; Secondly, participants in acupoints herbal patching group inevitably suffered much greater skin irritations, which will easily lead to the failure of blinding when participants from real acupoints herbal patching group communicated with placebo group; Thirdly, even participants have no idea of acupoints herbal patching before they entering this study, after joining this study, they might become interested in acupoints herbal patching and try to learn more about acupoints herbal patching. When participants know something about acupoints herbal patching, they might be easily aware that herbal patches consist of irritated ingredients which could incur skin irritations. Although blinding existed some inevitable limitations, its merits were still worth to be mentioned: firstly, herbal patch appearance look almost like placebo patch; secondary patch applied in the same 11 acupoints; thirdly, same duration and both in Sanfu Days. Even the Chinese Medicine practitioners have no idea about which group they conducted before unblinding. The nearly same drop-out rate in post-treatment and four times follow ups support that the blinding of this study was relatively successful. And after treatment, both groups got a significant improvement comparing with the baseline. All these show that placebo effect did exist during treatment and most participants in placebo group believed that they had received the real treatment.

β-agonists, which are the most common drug treatment during acute asthma attack while are associated with various adverse effects [4, 5]. In this study, the statistical results showed that the proportion of participants who didn’t need to use pharmaceutical treatment when they have a small rise in asthma-related symptoms was significantly increased after acupoints herbal patching. Besides, adverse events in this study were very minor, and the morbidity of side-effects in acupoints herbal patching group was insignificant higher than placebo group. TCM considerate that Sanfu Days are the three hottest in a year and both Yang-qi of body and nature are most exuberant in these days. From the temperature record of 2010, it was found that the average temperature in Sanfu Days from July 19, 29 and August 8 in 2010 (Table 5) was pretty high, all of them were above 31 °C, air humidity was above 77 %, and sunshine duration was more than 4.6 h [25, 26]. Hence, acupoints herbal patching in Sanfu days might be recommend as a complementary preventive treatment for chronic stable asthma especially for Qi-deficiency and Yang-deficiency patients.

## Conclusions

There was no significant difference between acupoints herbal patching and placebo group at the end of 3rd treatment and four times follow ups in pulmonary function test. The result was different from Li YH’s clinical studies about acupoints herbal patching for asthma, which show that acupoints herbal patching can significantly improve participants’ lung function [27]. Also, the secondary outcome of acupoints herbal patching group was no significant difference as compare with placebo group. However, the sub-analysis of secondary outcomes in the four times follow ups show that as time goes on, the lasting effect of acupoints herbal patching was superior to placebo especially in improving Lung-qi-deficiency and Spleen-qi-deficiency syndromes. What’s more, the lasting effect of acupoints herbal patching was better than placebo in reducing medication need when they had a small rise in asthma-related symptoms. Acupoints herbal patching will not only be a significant alternative treatment with a low risk of side effect for asthma participants, but also provide a good choice for health policy makers.

## Abbreviations

1st: first
2nd: second
3rd: third
4th: fourth
FEV1: forced expiratory volume in one second
FEV1/FVC (%): forced expiratory volume in one second/forced vital capacity
ITT: intention-to-treat
PP: per-protocol
SPSS: statistical Package for the Social Sciences
ANCOVA: analysis of covariance
FVC: forced vital capacity
SD: standard deviation
TG: treatment group
CG: control group
Nov: November
Feb: February
Ref: reference
TCM: traditional chinese medicine
RCT: randomized control trial
IRB: institutional review board
